# Light-field deep learning enables high-throughput, scattering-mitigated calcium imaging

**DOI:** 10.1073/pnas.2510337122

**Published:** 2025-11-25

**Authors:** Carmel L. Howe, Kate L. Y. Zhao, Herman Verinaz-Jadan, Pingfan Song, Samuel J. Barnes, Pier Luigi Dragotti, Amanda J. Foust

**Affiliations:** ^a^Department of Bioengineering, Imperial College London, Royal School of Mines, London SW7 2AZ, United Kingdom; ^b^Department of Electrical and Electronic Engineering, Imperial College London, South Kensington, London SW7 2AZ, United Kingdom; ^c^Faculty of Electrical and Computer Engineering, Escuela Superior Politécnica del Litoral, Guayaquil 090902, Ecuador; ^d^Department of Engineering, Information Engineering, University of Cambridge, Cambridge CB2 1PZ, United Kingdom; ^e^Department of Brain Sciences, Division of Neuroscience, United Kingdom Dementia Research Institute, Imperial College London, Hammersmith Hospital Campus, London W12 0NN, United Kingdom

**Keywords:** light-field microscopy, calcium imaging, two-photon imaging, neural circuits, deep learning

## Abstract

Light-field microscopy (LFM) enables high-throughput functional imaging by scanlessly encoding entire volumes in single snapshots. However, LFM’s computational burden and vulnerability to scattering limit its application to biological imaging. We present a light-field strategy for volumetric, scattering-mitigated neural circuit activity monitoring. A physics-based deep neural network, 2PiLnet, is trained with two-photon volumes and one-photon light fields. Light-field videos of jGCaMP8f-expressing neurons are acquired in neocortical brain slices. 2PiLnet reconstructs volumes with two-photon-like contrast and source confinement from scattered, blurry one-photon light fields from fields-of-view for which no two-photon images are provided. This enables automated segmentation and extraction of calcium signals with high signal-to-noise ratios and reduces optical crosstalk compared to conventional volume reconstruction methods. Imaging 100 volumes per second, we observe putative spikes fired at up to 10 Hz and the spatial intermingling of putative ensembles throughout 530×530×100-micron volumes. Compared to iterative algorithms, 2PiLnet workflows reduce light-field video processing times by several-fold, advancing the goal of real-time, scattering-robust volumetric neural circuit imaging for closed-loop and adaptive experimental paradigms.

## Significance

We leverage two complementary imaging modalities to capture neuronal activity in 3D brain tissues. Scanless light-field microscopy provides high speed and sensitivity, while two-photon scanning microscopy provides high contrast and is robust to light scattering. While previous strategies trade off these qualities, our deep-learning approach integrates them to detect activity with high speed, sensitivity, and contrast while minimizing the confusion of signals from different neurons. Our deep neural network generalizes to samples for which no two-photon images were viewed, enabling two-photon-like contrast and source confinement with simple, scanless one-photon optics. This speed and sensitivity can reveal how 3D neural circuits encode information through spike timing and how neuronal connectivity evolves.

Calcium fluorescence imaging enables observation of neuronal network function over time scales of milliseconds to months, and elucidates structure–function relationships through the simultaneous measurement of position, morphology, and activity (1). Although calcium provides a low-pass filtered proxy for neuronal spiking well sampled at a few hertz, certain applications benefit from sampling faster. For example, functional connectivity maps can be inferred from the correlation of calcium signals imaged at 5 Hz, but we cannot infer the connection polarity, or whether connections are mono- or poly-synaptic (2). Hence, next generation neural network monitoring must feature high speed, high sensitivity, and high throughput to capture the connectivity, dynamic states, and computations of living neural networks.

The development of fast, sensitive genetically encoded calcium indicators (GECIs) and new optical imaging technologies have enabled the study of functional neuronal networks in a variety of organisms, including mammals. However, the goal of fast, high-throughput, and volumetric imaging poses a particular challenge in mammals as our brains scatter light strongly. This is because the modalities providing the best optical sectioning and scattering mitigation, such as confocal and two-photon (2P) laser scanning microscopy, have a limited fluorescence bandwidth due to scanning (3). The finite bandwidth limits the speed and number of cells that can be simultaneously monitored, and numerous strategies have been developed to boost or efficiently exploit the fluorescence generated (4). Rapid acquisition from selected regions of interest (ROIs) obtains in serial through optimized (5–7) and rapid scanning and focusing (8–15), or parallelized through wavefront modulation (16–23). Scanned fluorescence excitation can be multiplexed and/or spatially extended into multifocal arrays (24–36), beads (37–39), lines (40–46), or planes (47–54). The added complexity of the above schemes generally limits their widespread use. Moreover, their bandwidth remains fundamentally limited to the extent of parallelization.

Light-field microscopy (LFM) (55) is a fully scanless volumetric imaging modality which maximizes fluorescence bandwidth. A microlens array (MLA) is placed at the native focal plane of a widefield microscope, and the camera sensor is conjugated to the MLA back focal plane. The microlenses confer kilo-scale stereovision, enabling reconstruction of an entire volume from a single snapshot called the light field (LF). LFMs for calcium imaging are easily implemented with off-the-shelf MLAs, widefield fluorescence excitation, and complementary metal-oxide semiconductor (CMOS) cameras. Volumetric LFM calcium imaging was first demonstrated in transparent zebrafish larvae (56–58). In the murine neocortex, LFM has achieved simultaneous recording of 1,000s of neurons in a ø4 × 0.2 mm volume at up to 18 Hz (59) and faster in smaller volumes (60). LFM’s maximal fluorescence bandwidth means that it will scale to image at faster rates and in more neurons thanks to faster, high pixel-count cameras and brighter, more sensitive calcium indicators.

Although LFM optical trains are simple and low-cost, to date, only a handful of neuroscience studies report their use. Two challenges hinder LFM’s widespread application to volumetric calcium imaging. The first is computational cost. Conventional model-based reconstruction of volumes from LFs relies on iterative 3D deconvolution (61–63). Recently data-driven deep-learning approaches (64–66), and physics-inspired deep neural networks (67, 68) have demonstrated fast, high-fidelity source localization and volume reconstruction in limited contexts. Certain approaches bypass volume reconstruction through phase space sparse coding (69), compressive sensing (70), or seeded-iterative demixing (SID) (60). The second challenge is that most tissues, including the mammalian brain, scatter light strongly, while conventional LFM volume reconstruction assumes ballistic propagation. Scanned confocal (71, 72), speckle (73), selective volume (74), and other structured illumination LFM implementations (75–77) mitigate scattered and out-of-focus fluorescence through spatial modulation of the excitation. Other strategies enhance signal extraction from scattering-corrupted LFs acquired with vanilla LFM optics through integration of scattering models (78, 79), compressive sensing (70), dictionary learning and convolutional sparse coding (42, 76), and iterative demixing (60). Structured illumination and postprocessing strategies mitigate scattering effects and enable calcium extraction up to ∼400 microns in the murine neocortex (60). Despite these advantages, LFM’s substantial computational burden precludes its application to activity-guided or closed-loop experimental paradigms (80).

Deep-learning approaches stand to overcome LFM’s computational burden and enable real-time processing (66, 81–83). One key challenge for deep learning is ensuring reliability. A notable example for calcium imaging is HyLFM proposed by Wagner et al. (65), which integrates LFM with light-sheet microscopy, with the latter providing ground truth for training and continuous validation. We recently introduced two physics-based deep-learning frameworks integrating the LFM wave-optics forward model and 2P scanned volumes to mitigate scattering effects while ensuring reliability of volume reconstruction (67) and source localization (68). Deep-learning integration of multimodal image acquisition can both mitigate scattering and accelerate calcium signal extraction.

Here, we introduce a rapid LFM calcium activity extraction approach exploiting physics-based deep neural networks (DNNs) trained with one-photon LFs and a 2P scanned z-stack (67). We segment the active neurons from 100-Hz LF videos and compute each neuron’s calcium time series. LFs are imaged in mouse brain slices coexpressing the structural marker tdTomato and jGCaMP8f, the fastest available GECI, in neocortical layer 2/3 excitatory neurons. We compare the signal-to-noise ratio (SNR) and neuron-to-neuron optical crosstalk of calcium activity extracted by our DNNs to those extracted from volumes reconstructed by conventional Richardson-Lucy (RL) deconvolution and by seeded-iterative demixing (SID) (60). Our pipeline extracts calcium activity from LF videos in FOVs for which the DNN has never viewed 2P volumes. Up to ∼60 active cells are detected simultaneously down to 100 microns below the slice surface. The 100 Hz LF capture and high SNR enable resolution of putative spikes fired at up to 10 Hz. Compared to other methods, calcium activity segmented from the DNN-estimated volumes feature similar SNR, reduced crosstalk, and reduced computation time. We show that putative neuronal ensembles, i.e. groups with similar calcium activity, spatially intermingle throughout the LF-encoded volumes. Our strategy thus advances the goal of fast, high SNR, real-time volumetric calcium imaging necessary for speed-sensitive functional connectivity mapping, adaptive and closed-loop experimental paradigms. Importantly, the DNN generalizes to FOVs for which no 2P ground-truth are provided, making it possible to exploit 2P-like scattering mitigation without the 2P microscope.

## Results

### 2PiLnet Enables Scattering-Mitigated Calcium Signal Extraction from One-Photon LF Videos.

**Fig. 1.**
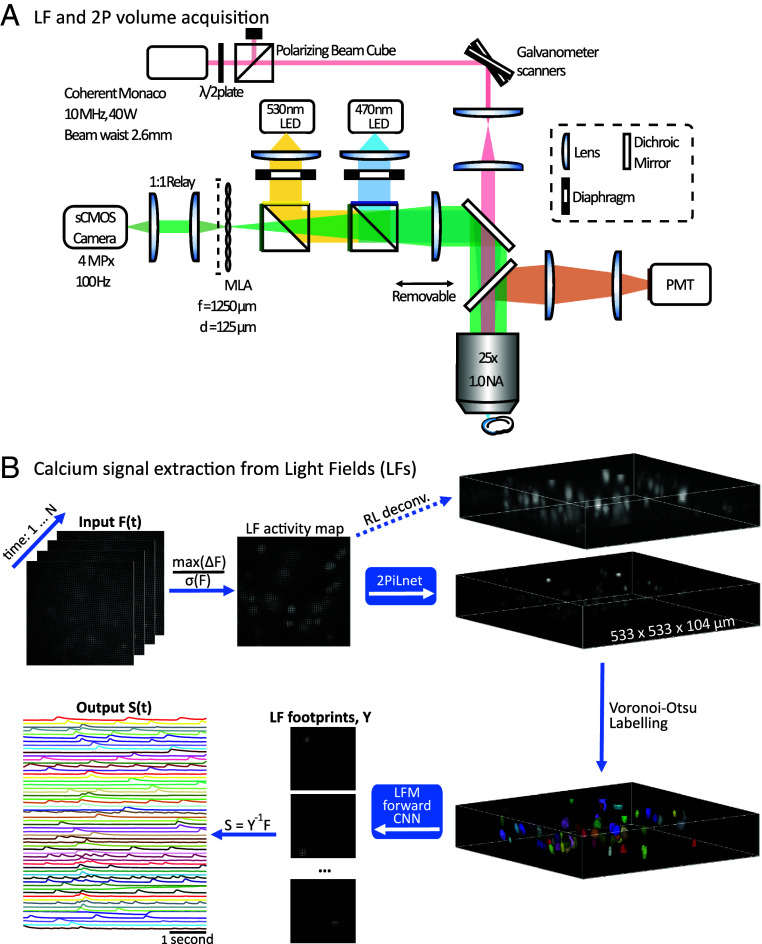
The 2PiLnet LFM and processing pipeline. (*A*) The dual-mode LFM combines a 2P laser scanning path with a one-photon LFM. A removable dichroic mirror reflects fluorescence toward the photomultiplier tube (PMT) for 2P collection. When the 2P dichroic is disengaged, fluorescence is imaged by the LFM with a microlens array (MLA). Distances not to scale. (*B*) Key elements of the deep-learning calcium activity extraction from one-photon LFs. LF videos (F) undergo pixel-wise preprocessing (max(ΔF)/σ(F)) to generate a single LF in which active neurons appear with high contrast. The LF activity map forms the input to 2PiLnet (67), which estimates a volume with 2P-like contrast and localization compared to the same volume computed via conventional 8-iteration Richardson-Lucy (RL) 3D deconvolution. Neurons are segmented from the 2PiLnet-estimated volume with 3D Voronoi-Otsu labeling. Each of the ROIs separately forms an input to a CNN computing the LFM wave-optics forward model and hence each neuron’s “footprint” in the light field. The vectorized footprints form matrix Y, and the calcium time series (S) are found as the product of the pseudoinverse of Y and F, the LF video.

**Fig. 2.**
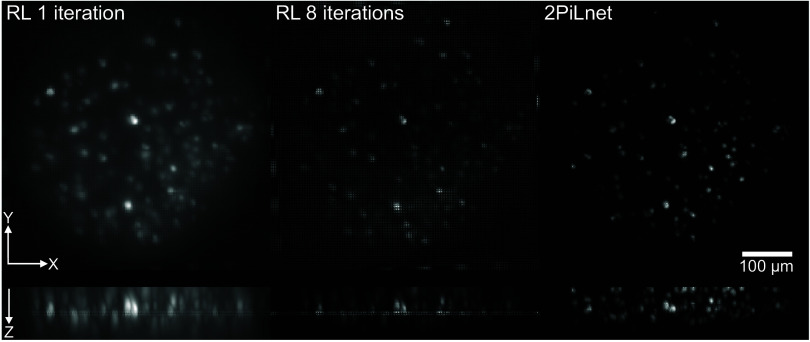
2PiLnet estimates volumes from one-photon light fields with 2P-like background suppression and source confinement. The panels display x-y and x-z maximum intensity projections of volumes reconstructed via 1-iteration RL (*Left*), 8-iteration RL (*Middle*), and 2PiLnet (*Right*) from a 100 Hz, five second LF video of soma-localized jGCaMP8f fluorescence. See also Movie S1.

LF images acquired in mammalian brain tissues are blurred by scattering and fluorescence generated outside of the LF-encoded volume. We recently introduced a 2P-informed and physics-based deep neural network (2PiLnet) which estimates scattering-mitigated volumes from one-photon LFs (67). 2PiLnet first undergoes supervised training with a 2P scanned volume and a coregistered one-photon light-field z-stack of layer 2/3 excitatory neurons transfected with the structural indicator tdTomato (Fig. 1*A*). 2PiLnet then undergoes self-supervised training with LF videos of the fast soma-localized calcium indicator ribo-jGCaMP8f (84). 2PiLnet integrates the LFM wave-optics forward model into the self-supervised training phase, which ensures reliable volume estimation. Indeed, good agreement is observed between the 2PiLnet output and the same volumes computed with purely model-based methods such as RL deconvolution (Fig. 1*B*, see also ref. 67). Maximum intensity projections of one-iteration RL-deconvolved volumes feature low contrast and poorly confined neurons, similar to widefield (85) (Fig. 2 and Movie S1). Contrast and confinement improve by increasing the RL deconvolution to eight iterations, but this also exacerbates the square-like artifacts around the native focal plane. Strikingly, the 2PiLnet-estimated volumes feature 2P image characteristics including high contrast, well-confined neurons on a dark background. In addition, the 2PiLnet volume is free of the square-like artifacts characteristic of RL-deconvolved volumes (63).

**Fig. 3.**
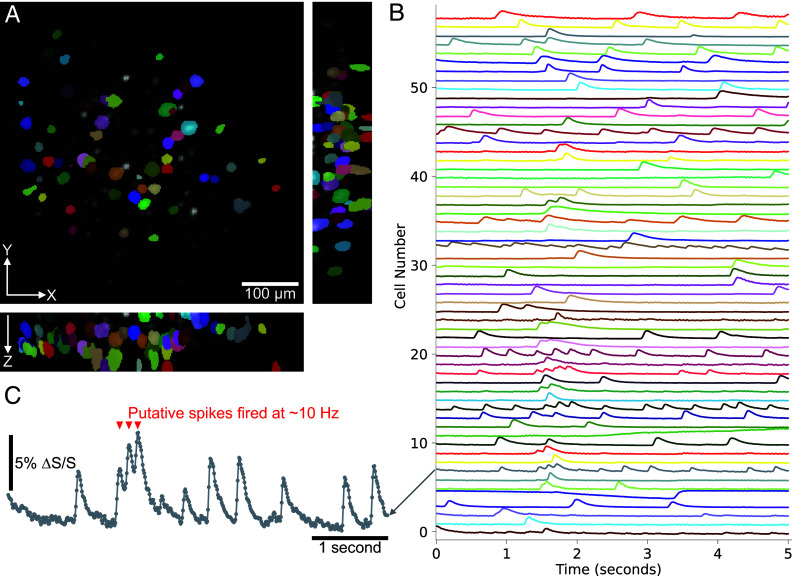
The 2PiLnet LFM enables the rapid extraction of high-SNR calcium transients from LF videos. (*A*) Shows the maximum intensity projection of volumes estimated by 2PiLnet from a 100 Hz, five second LF video. The overlaid ROIs are obtained with Veronoi-Otsu labeling of the volume estimated by 2PiLnet from the LF activity map. The ROI label colors correspond to the same-colored normalized time series obtained through matrix factorization displayed in (*B*). (*C*) shows an expanded view of one time series from (*B*). Red triangles indicate the time of putative spikes fired at a rate of ∼10 Hz. A Gaussian filter (σ=1) is applied to the displayed calcium time series.

We used 2PiLnet to systematically extract neuronal calcium activity from LF videos via two methods. Both methods begin with a pixel-wise calculation of the maximum change in fluorescence (ΔF) divided by the SD over time (σ(F)). The result is a single LF in which the active neurons appear with high contrast (Fig. 1*B*). This LF activity map forms the input to 2PiLnet. The 2PiLnet-estimated active neuron volume is segmented via Voronoi-Otsu labeling. In Method 1, called “2PiLnet region of interest (ROI),” 2PiLnet estimates volumes for each frame in the LF video. The calcium time series are then calculated as the mean intensity of voxels in each ROI of the activity map volume. In Method 2, called “2PiLnet Matrix,” each ROI forms the input to a convolutional neural network (CNN) computing the LFM wave optics forward model, hence estimating each active neuron’s footprint in the LF. The footprints are vectorized and assembled into a matrix Y. The calcium time series are then computed as the product of the pseudoinverse of matrix Y and F, the LF video. 2PiLnet LFM hence enables the rapid extraction of high signal-to-noise ratio (SNR) calcium transients from 100 Hz acquired LF videos encoding 533 × 533 × 104 micron volumes (Fig. 3). In this five second LF video, ∼60 active cells were detected in a volume containing over 150 neurons.

**Fig. 4.**
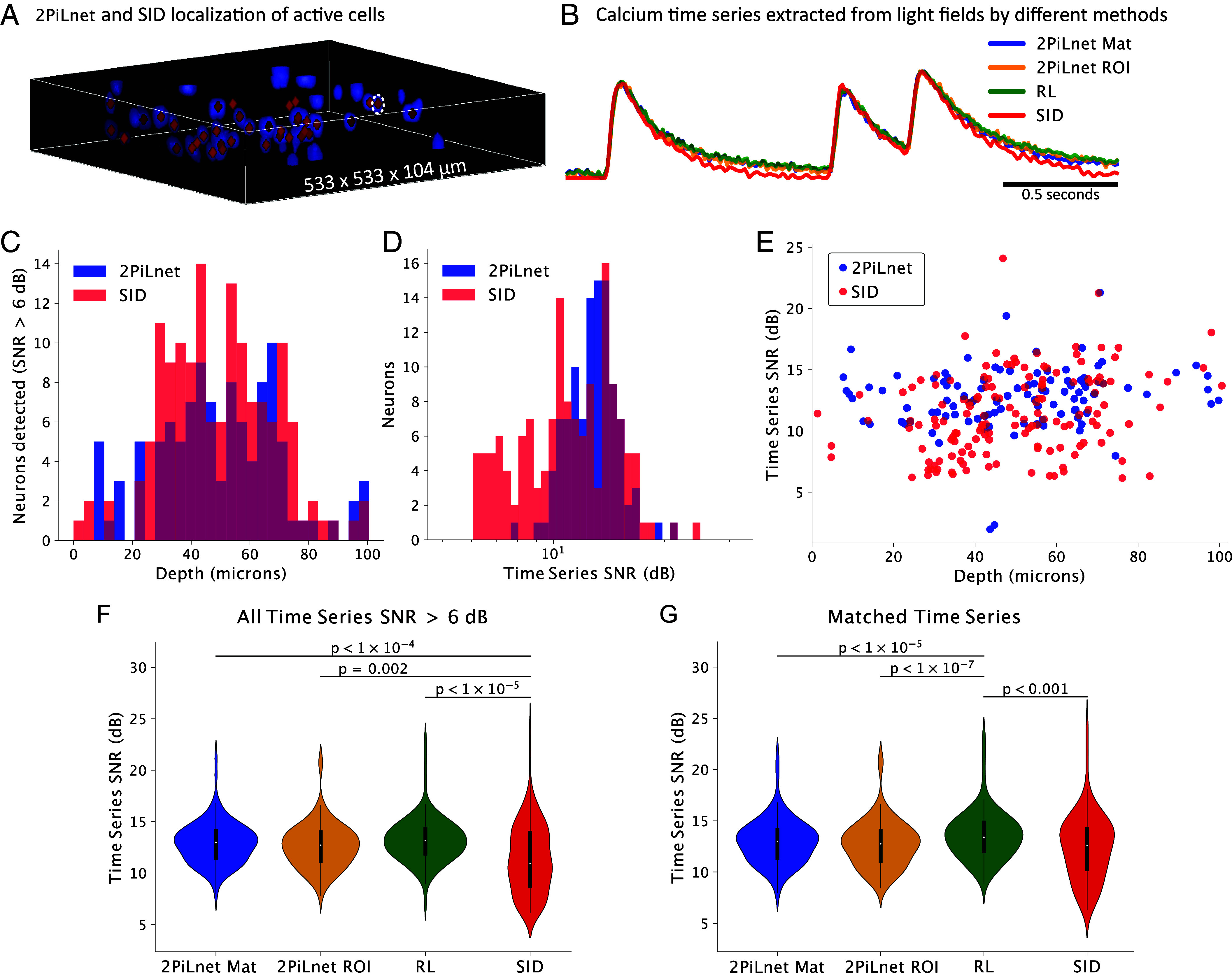
The 2PiLnet LFM detects high SNR calcium signals. The SNR of 2PiLnet-extracted calcium signals is benchmarked to conventional 8-iteration Richarson–Lucy (RL) deconvolution and seeded-iterative demixing [SID (60)]. (*A*) shows the 2PiLnet estimated activity map for a single ROI overlaid with 2PiLnet-segmented ROIs (blue) and neuron centroids localized by SID (red diamonds). (*B*) displays the normalized, unfiltered calcium time series for the neuron outlined with a dashed white line in (*A*), extracted via four methods: 1) 2PiLnet Matrix, 2) 2PiLnet ROI, 3) RL deconvolution, and 4) SID. (*C*) shows the number of neuronal calcium time series detected with SNR > 6 dB via 2PiLnet segmentation (blue) and SID (red) as a function of depth. (*D*) displays the histogram of SNRs for the same neurons (all depths) and (*E*) plots the SNR as a function of depth. (*F*) compares the SNR for all neurons with calcium time series SNR > 6 dB across methods (independent samples *t* test, *P*-values Bonferroni-corrected for multiple comparisons). (*G*) Displays paired comparisons of SNR across methods for “matched” time series in which neuronal centers are <20 microns apart and the Pearson correlation coefficient is >0.5 (paired samples *t* test, *P* values Bonferroni-corrected for multiple comparisons).

We benchmarked the SNR of time series extracted from 100 Hz jGCaMP8f LF videos via 2PiLnet ROI, 2PiLnet Matrix, conventional 8-iteration RL deconvolution, and SID. SID is a state-of-the-art algorithm developed specifically to mitigate scattering effects in LF calcium signal extraction (60). For RL, time series were extracted based on the 2PiLnet activity map ROIs, enabling paired comparison. As SID simultaneously localizes and demixes, the distribution of cells detected by 2PiLnet and SID differed, although with substantial overlap (Fig. 4*A*). The time series extracted via 2PiLnet and RL were similar for each ROI (Fig. 4*B* and *SI Appendix*, Fig. S1). The Pearson correlation coefficient of time series calculated for the same ROIs in the RL- and 2PiLnet-reconstructed volumes had a median of 0.99 and interquartile range (IQR) of 0.02 (n=107 cells). The high correlation with conventional model-based reconstructions reflects 2PiLnet’s reliability in this context. Both 2PiLnet and SID detected neurons throughout the 100-micron axial extent. 2PiLnet located 107 cells, and SID located 147 cells with time series SNR > 6 dB across five 250-frame LF videos acquired in five different FOVs (Fig. 4*C*). The 6 dB threshold excludes obvious false-positive time series with large noise fluctuations and no recognizable calcium transients (*SI Appendix*, Fig. S2). SID detected more cells with low SNR compared to 2PiLnet across all depths (Fig. 4 *D* and *E*). To assess true- and false-positive detection relative to ground truth, we simulated LF videos of neuronal calcium activity corrupted by scattering and noise (*SI Appendix*, *Supplementary Methods*). 2PiLnet featured higher precision and lower recall than SID under high Poisson noise conditions, indicating that SID recovers both more true- and false-positive time series (*SI Appendix*, Fig. S3).

We compared the SNR of time series extracted from the measured LF videos by 2PiLnet, RL, and SID. The mean time series SNR of 2PiLnet-localized cells was higher than SID-localized cells (Fig. 4*F*). The RL-derived time series had the highest SNR (median = 13.2 dB, IQR = 2.4 dB), followed by 2PiLnet Matrix (median = 13.0 dB, IQR = 2.6 dB), 2PiLnet ROI (median = 12.7 dB, IQR = 2.8 dB), and SID (median = 10.9 dB, IQR = 5.2 dB) across 5 FOVs obtained from 4 cortical slices in 3 mice (Fig. 4*F*). We identified matched time series for SID and 2PiLnet as cells with centers separated by less than 20 microns and time series correlation coefficients > 0.5 (n=75 neurons from five FOVs). The matched paired comparisons showed RL-derived time series SNRs significantly higher than those obtained with the other methods (as median, IQR in dB; RL: 13.4, 2.8; 2PiLnet Matrix: 13.0, 2.8; 2PiLnet ROI: 12.8, 3.0; SID: 12.6, 4.0; Fig. 4*G*).

### 2PiLnet Reduces Calcium Signal Cross-Talk in Neighboring Neurons.

**Fig. 5.**
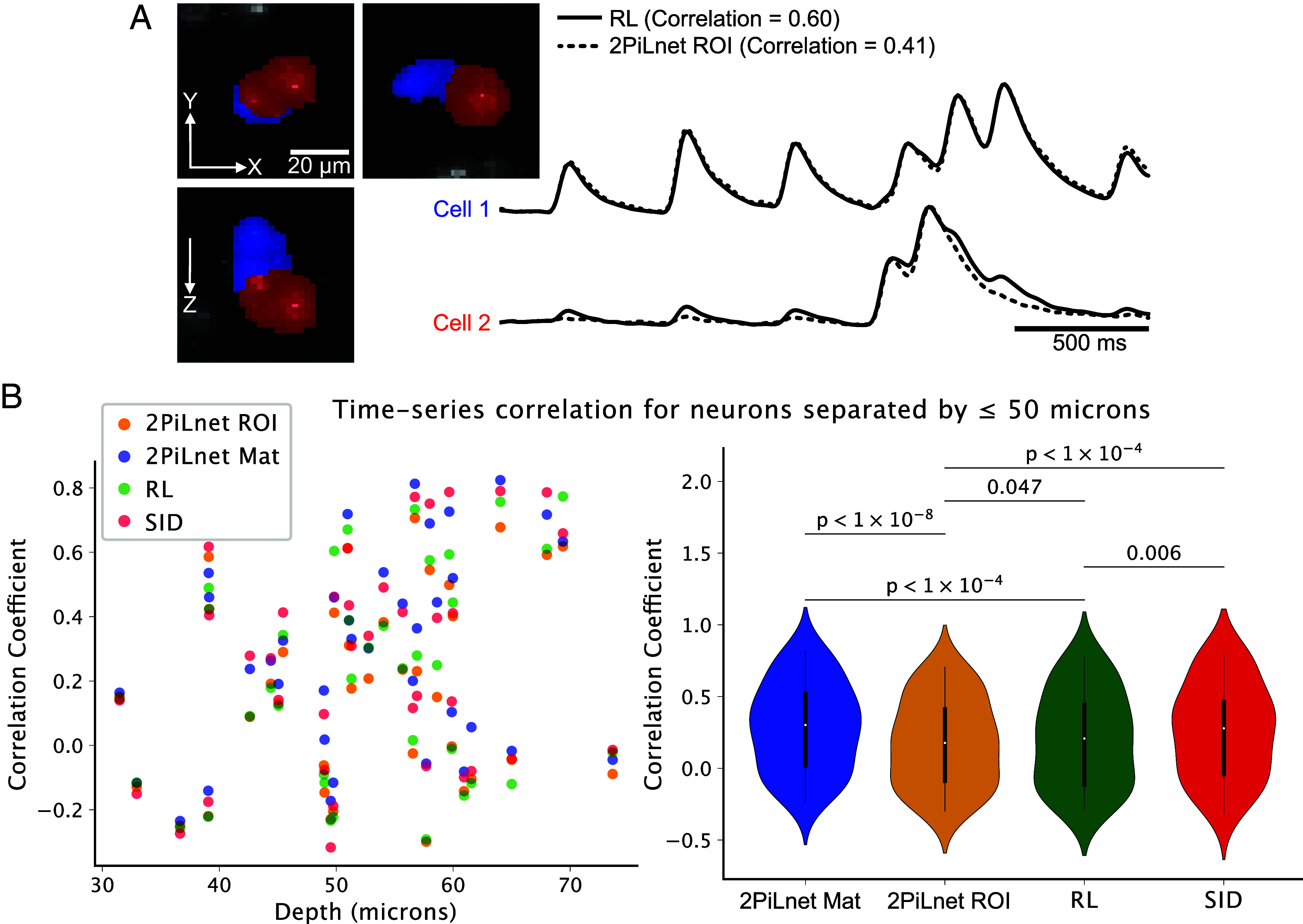
Calcium time series extraction from 2PiLnet-reconstructed volumes minimizes optical crosstalk. (*A*) Two cells segmented in the worst-case scenario for optical crosstalk: overlapping laterally and touching axially. The normalized time series extracted from these ROIs in RL-deconvolved volumes (solid black traces) are highly correlated (coefficient = 0.60) reflecting high crosstalk. Extracting the time series from the same ROIs in the 2PiLnet-estimated volume series (dashed black lines) decreases this correlation (coefficient = 0.41). A Gaussian filter (σ=2) is applied to the displayed calcium time series. (*B*, Left) Pearson correlation coefficients of calcium time series extracted via deep learning (“2PiLnet ROI,” “2PiLnet Matrix”), 8-iteration RL deconvolution, and SID. Time series were matched across methods, and the correlation between neighboring (centroids separated by ≤50 microns) neuron time series is plotted as a function of the mean pair centroid depth. (*B*, Right) Paired comparison of neighboring cell correlation coefficients for matched time series extracted via four methods (n=39 neighboring neuron pairs across five FOVs, paired samples *t* test, *P*-values Bonferroni-corrected for multiple comparisons).

Light scattering and insufficient optical sectioning cause crosstalk between calcium signals arising from adjacent neurons. Fig. 5*A* shows two axially adjacent cells, a worst-case configuration for optical crosstalk. Since point spread functions are extended in the axial dimension, signals arising from laterally overlapping and axially adjacent sources, such as the neurons indicated by the red and blue ROIs, are the most difficult to unmix. Indeed, the time series extracted from these neurons via conventional 8-iteration RL deconvolution of each volume in the LF video show substantial crosstalk (Pearson correlation coefficient = 0.60). Time series from the same ROIs in the 2PiLnet-estimated volumes show substantially less crosstalk, with a correlation coefficient of 0.41, two-thirds that of the RL-reconstructed volume series.

We benchmarked 2PiLnet’s neuron-to-neuron crosstalk against RL deconvolution and SID (60). We calculated the correlation coefficient of calcium signals from cells separated by a euclidian distance of ≤50 microns. Fig. 5*B* shows the correlation of matched time series as a function of the mean depth of the pair of neurons (n=39 neighboring neuron pairs from five FOVs). Time series from nearby neurons extracted with the 2PiLnet ROI method had lower correlation (median = 0.18, IQR = 0.50) than those extracted with the 2PiLnet Matrix method (median = 0.30, IQR = 0.50), RL (median = 0.21, IQR = 0.56), or SID (median = 0.28, IQR = 0.50; Fig. 5*B*). Although such correlations arise from a combination of optical crosstalk and true connectivity, the difference in correlation across these methods in matched adjacent neurons reflects differing levels of optical crosstalk. The 2PiLnet ROI-extracted time series featured the lowest crosstalk across mean pair depths up to 74 microns.

### 2PiLnet LFM’s Speed and Sensitivity Volumetrically Resolves Putative Spikes Fired at 10 Hz.

**Fig. 6.**
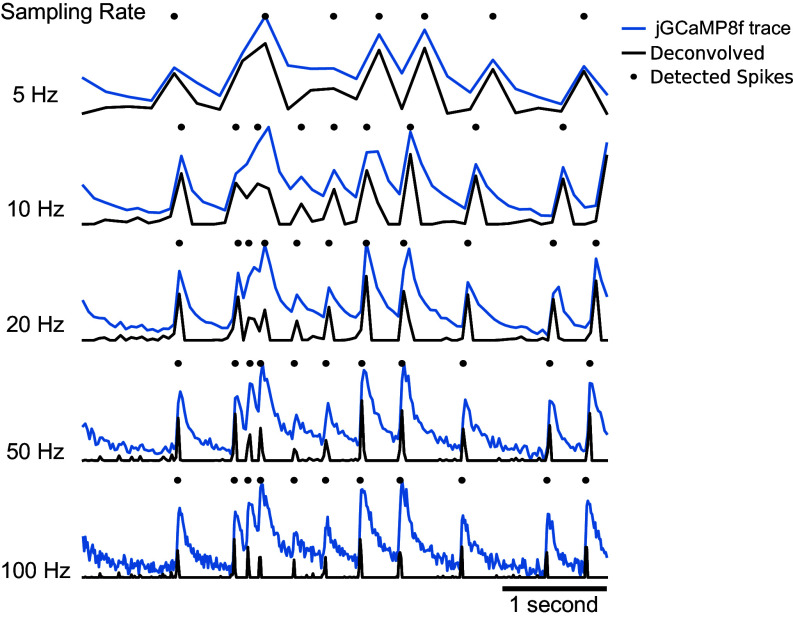
Deconvolution of neuronal calcium signals obtained with 2PiLnet LFM at 100 volumes/s enabling detection of putative spikes fired at 10-Hz rates. Example unfiltered jGCaMP8f calcium time series (blue traces) are deconvolved using the OASIS package (black traces) (86). Peaks in the deconvolved traces mark the timing of putative spikes (black dots). Downsampling by averaging adjacent time points boosts SNR but results in missed spikes and distorted spike timing.

We exploited 2PiLnet LFM’s bandwidth to track neuronal spiking dynamics reported by the fastest available GECI, jGCaMP8f (84), in volumes at 100 Hz. In our dataset, this enabled high SNR tracking of putative spikes fired at rates up to ∼10 Hz (Fig. 3*C*). Typically, volumetric and multiplane calcium imaging is performed at only a few Hz. Fig. 6 shows the impact of downsampling jGCaMP8f time series to 50 Hz, 20 Hz, 10 Hz, and 5 Hz. Here, we perform the downsampling by averaging, hence boosting the SNR for lower rates by the square root of the downsampling factor. We detected putative spikes as peaks in the deconvolved calcium traces (86). Even though averaging boosts the 5 Hz and 10 Hz time series SNR by three to fourfold, putative spikes are missed and the timing of detected spikes distorted.

### Neuron Ensembles Spatially Intermingle in LF-Encoded Volumes.

**Fig. 7.**
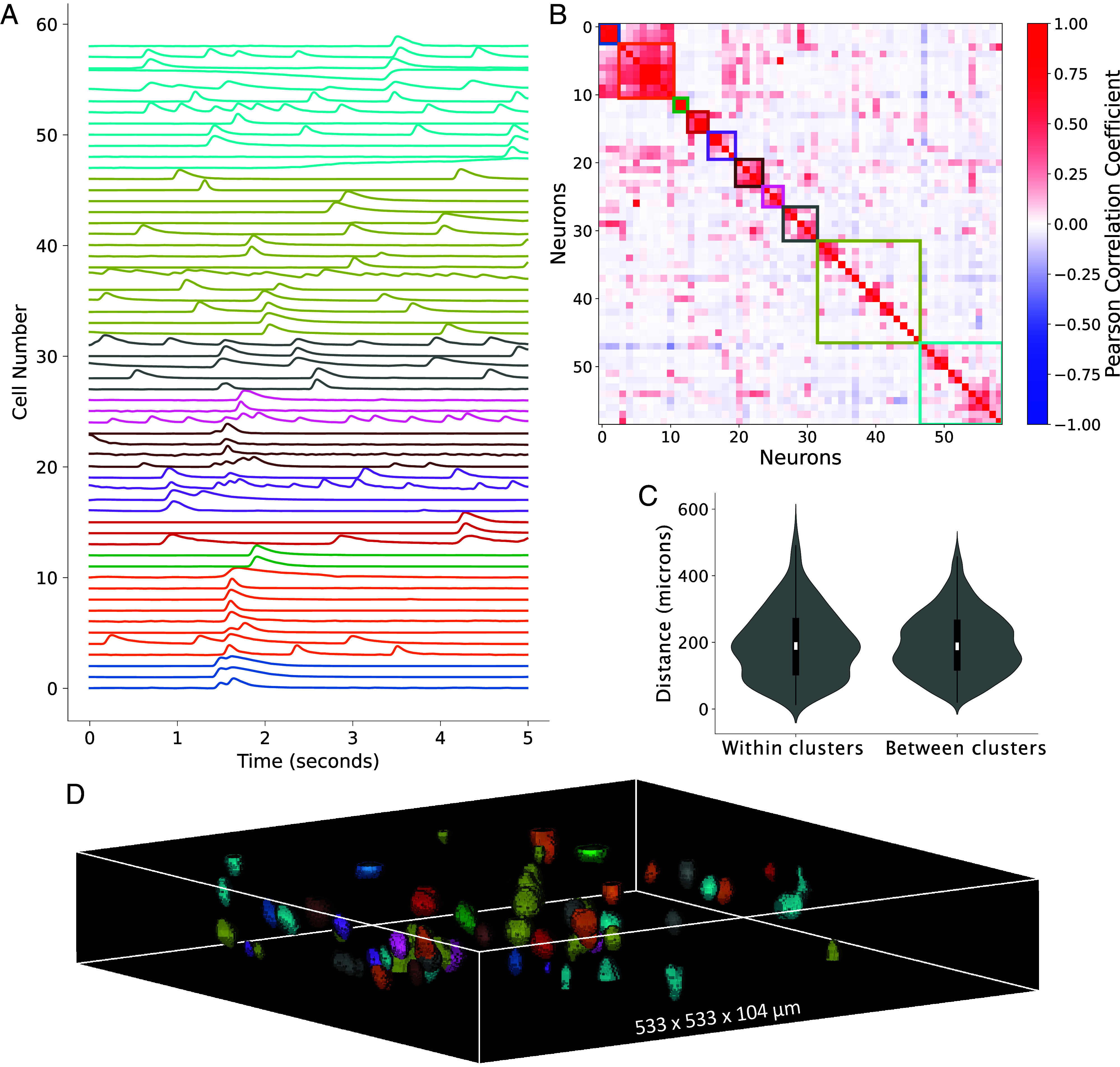
Neurons with correlated calcium activity intermingle in 2PiLnet LFM-encoded volumes. (*A*) Calcium time series and correlation matrix (*B*) of the deconvolved traces (Fig. 6) ordered and grouped with agglomerative hierarchical clustering. The color-coded time series and boxes on the correlation matrix (*B*) indicate the clusters. A Gaussian filter (σ=2) is applied to the displayed calcium time series. (*C*) The euclidian distance between cells within and between clusters from five FOVs was not statistically different (P>0.5 for Mann–Whitney *U* and independent samples *t* tests). (*D*) visual inspection of color-coded ROIs in the volume shows spatial intermingling of neurons from different clusters.

The high speed and SNR of 2PiLnet LFM can be exploited to map neuronal ensembles in 3D. We performed agglomerative hierarchical clustering on the deconvolved 100-Hz calcium time series extracted with 2PiLnet from each of five FOVs. Fig. 7 shows the calcium time series (*A*) and Pearson correlation coefficients (*B*) for the clustered deconvolved calcium signals extracted from one FOV. The euclidian distance between cells within a cluster (median = 191 microns, IQR = 158 microns, n=579 distances) and between clusters (median = 193, IQR = 137 microns, n=2,079 distances) was not statistically different (Fig. 7*C*). Indeed visual inspection of the segmented cell clusters in the example FOV shows that cells with correlated activity spatially intersperse among cells from other groups throughout the volume (Fig. 7*D*).

### 2PiLnet Reduces Single-Workstation LF Video Processing from Hours to Minutes.

The computational load and complexity of volume reconstruction from LFs has previously hindered LFM’s application to neurophysiology and biological imaging in general. Nöbauer et al. previously demonstrated that SID reduced computational resource requirements by three orders of magnitude compared with conventional LFM reconstruction (e.g. iterative 3D deconvolution such as RL), bringing LFM calcium videos processing down to the capacity of a single workstation.

We compared the computation time required for 2PiLnet-based calcium time series extraction with that of SID on a single workstation equipped with a NVIDIA GeForce RTX 2080 Ti graphical processing unit (GPU). Calcium time series extraction with SID took ∼12 h per 250-frame LF video. Initial training of 2PiLnet took ∼20 h (400 epochs at ∼3 min/epoch). Following this one-time training, 2PiLnet was applied to jGCaMP8f videos in previously unseen FOVs with brief fine-tuning taking ∼30 min (10 epochs at ∼3 min/epoch) with the LF activity maps for the 2PiLnet Matrix workflow. For the 2PiLnet ROI method, 2PiLnet was fine-tuned simultaneously on every sixth frame of the five LF videos for 100 epochs. This fine-tuning took 300 min or five hours (100 epochs at ∼3 min/epoch). Subsequent LF preprocessing, volume reconstruction, segmentation, and time series extraction, either via footprint estimation and matrix factorization (2PiLnet Matrix) or by averaging intensities from the 2PiLnet-estimated volume series (2PiLnet ROI) took only a few minutes. Although the initial computationally greedy 2PiLnet training takes several hours, subsequent calcium signal extraction from unseen FOVs using the 2PiLnet Matrix workflow takes under 40 min, including fine-tuning, on a single workstation. 2PiLnet ROI required more time for fine-tuning but reduced optical crosstalk compared to all methods tested.

## Discussion

In this article, we have introduced physics-based deep-learning strategies for high sensitivity, scattering-mitigated volumetric calcium signal extraction from LF videos. The videos were acquired at 100 Hz, enabling high-speed volumetric capture of neural dynamics reported by the fast GECI jGCaMP8f. 2PiLnet, trained on a single 2P volume stack and fewer than 300 one-photon LFs, conferred 2P-like contrast and source confinement to volumes estimated from LFs encoding previously unseen FOVs. The 2PiLnet LFM enabled extraction of calcium time series within a ∼530 × 530 × 100 micron volume in mouse neocortical brain slices. Ensembles of neurons with similar calcium time series spatially intermingled throughout the volumes.

One primary challenge with deep learning is ensuring reliability. DNNs can hallucinate features from noise. The integration of the wave-optics forward model into 2PiLnet’s architecture protects against unrealistic volume estimation. Indeed, time series extracted from the same ROIs in the 2PiLnet- and RL-reconstructed volumes were highly correlated (median coefficient 0.99) indicating high reliability.

2PiLnet LFM resolved putative spikes fired at rates up to 10 Hz. Its high volume rate and SNR, combined with jGCaMP8f’s fast decay (84), can improve tracking of complex spike events and of interneurons which produce smaller calcium transients, higher spike rates, and less bursting than excitatory neurons (87). Moreover, the 100 Hz volume rate captured 3 to 4 points on the fast rising phase of each calcium transient (Fig. 3*C*), adequate to infer connection polarity with 10 ms precision. The volume rate was limited by the camera’s maximum full field frame rate. Faster, more sensitive and low-noise sCMOS cameras are already commercially available, which would enable even faster volume rates with an approximately 1.5 dB SNR loss per speed doubling all other factors being equal.

Compared to scattering mitigation strategies based on scanned or structured illumination (3), scanless 2PiLnet LFM stands out because of its maximal fluorescence bandwidth, speed (100 volumes/s), and sensitivity (median SNR ∼13 dB). Compared to 2PiLnet, the SID algorithm detected more time series with SNR <10 dB than 2PiLnet. Time series recovered by SID from simulated LF videos consisted of both true and false positives, whereas 2PiLnet recovered only true positives. In measured LF videos, SNR was similar for time series detected by both strategies. While 8-iteration RL achieved the highest SNR of all methods, it had worse optical crosstalk than the 2PiLnet ROI pipeline. We used ROIs segmented from the 2PiLnet-estimated activity volume to extract the time series from the RL-reconstructed volumes. Hence, this comparison likely quantifies the best case for RL crosstalk. In practice, high background and poor source confinement make it difficult to segment the RL-reconstructed volumes on their own.

One striking feature of 2PiLnet is the ability to confer 2P-like contrast and source confinement to FOVs for which it only “sees” one-photon LFs. Indeed, time series extracted from the same neighboring neuron pairs from the 2PiLnet-reconstructed volumes were less correlated than those extracted from RL-reconstructed volumes, implying reduced optical crosstalk. The 2PiLnet ROI method also reduced crosstalk compared to SID, which discriminates calcium activity for neurons separated by distances down to 20 microns according to ground truth 2P imaging (60). Indeed, minimizing optical crosstalk between nearby neurons is critical for the accurate characterization of fine spatial scale functional motifs such as microclusters (88).

Following the one-off computationally greedy 2PiLnet training, 2PiLnet can reliably extract calcium time series from previously unseen FOVs following brief fine-tuning. The entire 2PiLnet Matrix pipeline, including fine-tuning, takes under 40 min on a single workstation, an 18-fold reduction compared to SID processing of the same videos. Fine-tuning for the 2PiLnet ROI pipeline took ∼5 h and achieved the lowest optical crosstalk. There is scope to reduce the time needed for fine-tuning by, e.g., keeping only a few layers trainable or adjusting the loss function. While end-to-end the 2PiLnet Matrix pipeline computed eightfold faster than the 2PiLnet ROI pipeline, the latter reduced crosstalk while maintaining similar SNR. Hence, the 2PiLnet ROI method is better suited to applications sensitive to crosstalk between densely labeled neurons. In contrast, the 2PiLnet Matrix method is better suited to sparsely labeled samples and to scenarios limited by time and computing power. Further adaptation of 2PiLnet to GPU and field-programmable gate array (FPGA) architectures (89) may soon enable real-time signal extraction for adaptive and closed-loop neuroscience experiments.

In this study, we imaged the most superficial 100 microns of submerged neocortical brain slices, which contained the well-perfused active cells. Future studies will assess 2PiLnet’s performance at greater depths in-vivo. In-vivo imaging will require correction of motion artifacts not encountered here in-vitro. For motion correction, widefield LFMs have a significant advantage over scanned or structured illumination strategies where motion can cause complete signal loss as sources shift out of the excitation pattern. Widefield LFMs excite and encode fluorescence simultaneously throughout the volume which can be aligned post hoc.

The initial training of 2PiLnet was performed with a single 2P z-stack. Application to other FOVs required no additional 2P data. It is conceivable that 2PiLnet could also process light fields acquired by other microscopes with analogous LFM optics without the costly 2P laser, scanning, and collection optics. Our particular LFM’s resolution and ∼530 × 530 × 100 micron FOV were determined by our microscope objective and MLA, but in principle bespoke versions of 2PiLnet can be trained for a variety of LFMs, including mesoscale (59) and head-mounted miniscope (90) configurations, given a small set of images acquired in both 2P and one-photon LFM modes. The advantage is that, following the appropriate one-off training, end users would only need simple LFM optics and a single work station. This would enable access to high-throughput, scattering-mitigated, volumetric imaging capability with simple hardware similar to widely used widefield imaging systems like Inscopix. A critical next step to realizing this vision is characterizing 2PiLnet’s generalizability to different LFM optics, including system-to-system alignment variation, and to samples expressing different indicators at different sparsities and depths.

## Materials and Methods

### The Dual Mode One-Photon LF and 2P Laser Scanning Microscope.

The home-built upright microscope collected 2P scanned images and one-photon epifluorescence LFs in the same FOVs (Fig. 1*A*).

For 2P imaging of tdTomato, a large removable dichroic mirror (50 × 70 × 2 mm, T750lpxrxt-UF2, Chroma) engaged directly above the objective (25×, NA 1.0, XLPLN25XSVMP, Olympus) back aperture, which was conjugated and demagnified onto the active area of a photomultiplier tube module (PMT, Hamamatsu H10722-20-10MHz). The tdTomato fluorescence was shortpass filtered with a 750-nm cutoff (Semrock FF01-750/SP-25). The PMT was powered by a programmable DC power supply (Radiospares, RSPD3303C) controlled by a custom graphical user interface GUI written in Python 3. A Mini-circuits BLP-1.9+ (2.5 MHz cutoff) low-pass filtered the PMT module output voltage. The conditioned PMT signal was digitized by a National Instruments PCI-6110 and assembled into images and z-stacks using ScanImage version 3.8 software. ScanImage also controlled the scan engine through two PCI-6110 analog outputs to high-power servo boards (671315K-1HP, Cambridge Technologies) driving three-millimeter galvanometric mirrors (6M2003S-S, Cambridge Technologies) which laterally scanned the beam of a Coherent Monaco amplified fiber laser (Coherence Monaco 1035-40-40, center wavelength 1035 nm, pulse width 286 fs, frequency 10 MHz, beam waist 1/e^2^ 2.6 mm, 5 to 10 mW power under the objective). A 30-mm focal length scan lens and a 300-mm focal length tube lens conjugated and expanded the beam at the galvos onto the objective back aperture. A dichroic mirror (DI03-R785-T3 25 × 36 × 3 mm, Semrock) between the tube lens and the 2P collection dichroic coupled the beam into the optical train shared with the one-photon LFM path.

For the LFM, one-photon fluorescence was excited in widefield with light-emitting diodes (LEDs) conjugated to the objective back aperture with 35-mm focal length plano-convex lenses and a 180-mm tube lens (TTL180-A, Thorlabs). TdTomato fluorescence was excited with a 530 nm LED, and jGCaMP8f fluorescence was excited by a 470 nm LED, both powered by an OptoLED current driver (P1110/002/000, Cairn Research). Semrock FITC-Ex01-Clin (excitation), Semrock FF495-DI03 (dichroic), and Semrock FF01-550/88 (emission) formed the jGCaMP8f filter set. Semrock FF01-520/35 (excitation), Semrock FF552-Di02 (dichroic), and Chroma ET605/52m (emission) formed the tdTomato filter set. The objective and the tube lens imaged the native focal plane onto an off-the-shelf microlens array (MLA, 125 µm pitch, f/10, RPC Photonics). A 1:1 macrolens (Nikon 60 mm f2.8 D AF Micro Nikkor Lens) relayed the MLA back focal plane onto the scientific CMOS camera (ORCA Flash 4 V2 with Camera Link, 2,048 × 2,048 pixels, 6.5 µm pixel size, Hamamatsu).

The LFM native lateral resolution is approximated by the microlens pitch (125 µm) divided by the objective magnification (25×), or 5 µm. The axial resolution of a LFM is determined by the number of resolvable diffraction-limited spots behind each microlens (55). Using the Sparrow criterion and assuming an average emission wavelength of 530 nm, the spot size in the camera plane is 6.2 µm, slightly smaller than the 6.5-µm sCMOS camera pixels. Hence, given the 125-µm MLA, we are able to resolve ∼19 distinct spots under each microlens. This corresponds to a synthetically refocusable depth-of-field of 7.5 µm over a 131-µm axial range. The deconvolved resolution generally exceeds the above in a depth-dependent fashion outside of the native focal plane where angular information is redundant and square-like artifacts dominate (63).

LF videos of jGCaMP8f-expressing neurons were imaged at 100 frames/s using Micromanager 2.0-gamma (91). A stepper motor (SliceScope, Scientifica) controlled by ScanImage and Micromanager scanned the objective along the optic axis to acquire 2P and LF z-stacks of tdTomato-expressing neurons.

### 2PiLnet Structure and Training.

We recently introduced 2PiLnet as a means to exploit the scattering mitigation of 2P laser scanning microscopy to estimate low-background, well-sectioned volumes from blurry one-photon LFs. Full details of 2PiLnet’s architecture and training can be found in ref. 67. Briefly, a filter bank representation of the LFM wave-optics forward model is expressed as a CNN. We formulate the inverse problem, inferring volumes from LFs, as an optimization promoting sparsity in the reconstruction, since the compact jGCaMP8f somata occupy a small fraction of the total reconstructed volume. The optimization is solved using the Iterative Shrinkage-Thresholding Algorithm whose iterations are unfolded into the layers of a CNN. The parameters of 2PiLnet are trained in two phases. The first phase consists of supervised training with a LF z-stack (28 images with a 2-micron z-step) and a 2P z-stack (80 planes with a 2-micron z-step) of the same tdTomato expressing neurons in a cortical brain slice. Each light field in the z-stack is paired with a 2P substack consisting of 53 depths, 2-micron step, vertically centered on the LFM native focal plane. This results in 28 LF/2P-volume pairs which form the labeled dataset for supervised training. The second training phase is self-supervised, since there are no ground truth 2P volumes for the jGCaMP8f LF videos. Here, the LFM forward model CNN computes LFs from volumes estimated by 2PiLnet, and the loss between the computed and input LFs is minimized. The LF loss is regularized by an adversarial loss consisting of a critic trained to distinguish between real and false volumes based on the 2P z-stack. The self-supervised training was conducted with three 80-frame jGCaMP8f videos acquired at 50 frames/s from three different FOVs.

In total, 2PiLnet was trained with very little data: 108 structural images (a 28-image LF z-stack and an 80-image 2P z-stack from a single FOV) and 240 jGCaMP8f LF video frames (three 80-frame, 50 Hz videos). Impressively, 2PiLnet generalizes to previously unseen FOVs, for which there is no 2P ground truth, including the full dataset presented here, with only brief fine-tuning. For reconstructing the five LF activity volumes, fine-tuning was run for 10 epochs using five LF activity maps calculated from the five LF videos. For the 2PiLnet ROI pipeline, we fine-tuned 2PiLnet for 100 epochs on every sixth frame of the five LF videos simultaneously. Importantly, fine-tuning retains the original training data while incorporating only the new jGCaMP8f LF images, without requiring additional modalities or labeled datasets.

### jGCaMP8f Time Series Extraction from LF Videos.

Our time series extraction approach starts with preprocessing of jGCaMP8f LF videos to generate a single LF activity map in which active neurons appear with high contrast (Fig. 1*B*). First, a size three uniform filter is applied to each pixel across time. Max(ΔF) is calculated in each pixel and divided by the SD of the unfiltered time series, σ(F). The Max(Δ F)/σ(F) array is then multiplied by a factor of 1,024 and the median pixel value calculated. The difference of the median of the scaled array and 16 is subtracted from each pixel. Then all values are truncated to a maximum value of 150 (such that higher values are set to 150) and a minimum value of 9, corresponding to the camera dark value on an 8-bit scale. These preprocessing steps make active neurons appear with high contrast in the activity map while keeping the minimum, maximum, and median values similar to those of the jGCaMP8f 2PiLnet training videos. LFs were rectified and formatted in Matlab with a custom script.

The LF activity map forms the input to 2PiLnet which estimates the active neuron volume of size 231 × 231 × 53 voxels covering a 533.3 × 533.3 × 104-micron volume. The activity map volumes are segmented with the Voronoi-Otsu labeling method (py-clEsperanto prototype, $\sigma_{\mathrm{spot}}=3$ and $\sigma_{\mathrm{outline}}=1$). From this point, the 2PiLnet ROI and 2PiLnet Matrix pipelines diverge. For 2PiLnet ROI, 2PiLnet is fine-tuned on every sixth frame of the LF video and then reconstructs the entire volume series. Time series are calculated as the mean intensity of pixels across time for each ROI in the segmented activity volume. For 2PiLnet Matrix, each segmented ROI is put into an 8-bit volume containing zeros everywhere except for voxels within the ROI which are set to a value of 150. Each ROI volume is then fed to the LFM forward model CNN which computes each neuron’s contribution, or “footprint,” in the LF. The neuronal LF “footprints” are flattened and assembled into a matrix Y. The calcium time series (S) are then obtained through spatiotemporal factorization: $S=Y^{-1}F$, where F is the LF video and $Y^{-1}$ is the pseudoinverse of the rectangular LF footprint matrix. The 2PiLnet processing pipelines are implemented in Python 3.

### Implementation of Benchmark Methods.

We benchmarked the performance of the 2PiLnet Matrix and 2PiLnet ROI methods against that of conventional model-based 3D RL deconvolution and SID (60).

For RL, we reconstruct each frame individually to obtain a volume time series. We used 8 iterations with the backprojection volume from the transpose operation as the initialization. Time series were calculated as the average intensity of each ROI across the volume time series, using the ROIs segmented from the 2PiLnet activity map to enable direct comparison. RL deconvolution was performed in Python 3 using PyTorch.

For SID, we used the official SID implementation in MATLAB (60). The algorithm starts with background subtraction (2 iterations, detrending disabled) and computes a SD image from the background-subtracted data. Initialization is then performed using nonnegative matrix factorization with a rank of 15, an L1 norm regularization of the Gram matrix set to 1e-3, and 600 iterations. Cross-validation was enabled using 7 partitions. Each spatial component from the factorization is then reconstructed to obtain a volume, which is further segmented to extract neuron candidate positions. The reconstruction uses Projected Gradient Descent with Exact Line Search running for 3 iterations. L1 regularization was set to 0.1, and total variation regularization was applied with values 0.1, 0.1, and 4. Kernel optimization was disabled, and filtering was applied. The segmentation step was performed using a threshold of 0.1, with top and bottom cutoffs set to 5 and 49, respectively, and a neuron radius of 20 pixels. Cropping was not applied. All other parameters were set to their default values. Finally, the core of the SID algorithm consists of an iterative process alternating between spatial and temporal updates, refining both the temporal signals and spatial footprints, which constitute the detected neurons.

### Data Analysis.

We compared SNR, which quantifies sensitivity to changes in calcium indicator fluorescence, across methods and as a function of depth below the slice surface. For the SNR calculation, the z-score of the time series was smoothed with a size 10 uniform filter. Noise (N) was calculated as the SD of the difference between the smoothed and unfiltered traces. The signal (S) was the difference of the maximum and minimum values of the smoothed time series. SNR was calculated by taking the ratio and converted to a dB scale: $SNR_{\mathrm{dB}}=10*\log_{10}\left(S/N\right).$ Due to differences in scaling and offset, ΔF/F cannot be directly compared across methods. The vertical scale in Fig. 3*D* is expressed as $\Delta S/S=\frac{S(t)}{{\left\langle S\right\rangle }_{T}}-1$, where ${\left\langle S\right\rangle }_{T}$ is the mean value of the extracted time series.

Time series were deconvolved using the OASIS algorithm (86) in the suite2P python module (92). Time series correlations were measured using the Pearson correlation coefficient. Hierarchical agglomerative clustering on the deconvolved calcium time series sorted the neuronal responses into groups with similar activity. For each FOV the number of clusters was chosen based on the number of detected active neuron time series such that the average number of time series per cluster was ∼6.

LF processing and analyses were carried out in Python 3. Volumes and videos were visualized and rendered with napari (93).

### Transfection and Brain Slice Preparation.

The following protocols were previously described in ref. 67 and summarized below.

This study was carried out in accordance with the recommendations of the UK Animals (Scientific Procedures) Act 1986 under Home Office Project and Personal Licenses (project license 70/9095). Mouse layer 2/3 cortical neurons were transfected by in-utero electroporation (IUE) at embryonic day (E)15.5 using soma-targeted jGCaMP8f [pAAV-CAG-RiboGCaMP8f (84, 94)] and tdTomato [pCAG-tdTomato, Addgene (95)]. Time-pregnant female CD-1 mice (Charles River UK) were deeply anesthetized with 2% isoflurane. The uterine horns were exposed and intermittently rinsed with warm sterile phosphate-buffered saline (PBS). A plasmid mixture totaling 1 to 2 µg at a concentration of 1 µg/µl (6:1 ratio of jGCaMP8f:tdTomato), diluted in sterile PBS, was injected into one of each embryo’s lateral ventricles. Five electrical pulses (50 V, 50 ms each, at 1 Hz) were applied using 5-mm round plate electrodes (ECM 830 electroporator, Harvard Apparatus) with the anode positioned over the skull to target the cortex. Electroporated embryos were returned to the mother to continue development until birth.

Brain slices from electroporated mice were made at postnatal day (P)12-P30. 400-micron thick slices were cut in choline chloride solution containing (in mM): 110 choline-Cl, 25 NaHCO_3_, 20 glucose, 2.5 KCl, 1.25 NaH_2_PO_4_, 0.5 CaCl_2_, and 7 MgSO_4_. The slices were transferred to an artificial cerebrospinal fluid (ACSF) solution containing (in mM) 125 NaCl, 25 NaHCO_3_, 20 glucose, 2.5 KCl, 1.25 NaH_2_PO_4_, 2 MgSO_4_, 2 CaCl_2_, adjusted 300 to 310 mOsm/kg, pH 7.3 to 7.4 with HCl at 36C. Slices rested in ACSF for at least 20 min prior to transfer to a submersion chamber perfused with the same solution at room temperature throughout the imaging session. All solutions were oxygenated with 95% O_2_/5% CO_2_. Some slices were perfused with 50 µM 4-aminopyridine to evoke robust activity during imaging.

## Supplementary Material

Appendix 01 (PDF)

Movie S1. Movie of 2PiLnet (right), 8-iteration RL deconvolved (middle), and 1-iteration RL deconvolved (left) volumes, related to Figure 2. Playback at 0.5x real speed.

## Data Availability

The raw LF videos and 2PiLnet training weights are deposited at Zenodo (DOI: 10.5281/zenodo.14900715) (96). All original code for the end-to-end 2PiLnet Matrix and 2PiLnet ROI systems is publicly available at https://github.com/afoust/LNet_jGCaMP8f_pipelines.
