# Supplementary material for: Light-field deep learning enables high-throughput, scattering-mitigated calcium imaging
Source: Proc Natl Acad Sci U S A. 2025 Nov 25;122(48):e2510337122. doi: 10.1073/pnas.2510337122 (PMC12685042; doi:10.1073/pnas.2510337122)
Supplement: Supplementary file 1 — Appendix 01 (PDF) [file pnas.2510337122.sapp.pdf]

Supporting Information for

Light-field deep learning enables high-throughput, scattering-mitigated calcium imaging

Carmel L. Howe, Kate L.Y. Zhao, Herman Verinaz-Jadan, Pingfan Song, Samuel J. Barnes, Pier Luigi Dragotti, Amanda J. Foust

Amanda J. Foust
Email: a.foust@imperial.ac.uk

This PDF file includes:

Figures S1-S3
Supplementary Methods
Legend for Movie S1

Other supporting materials for this manuscript include the following:

Movies S1

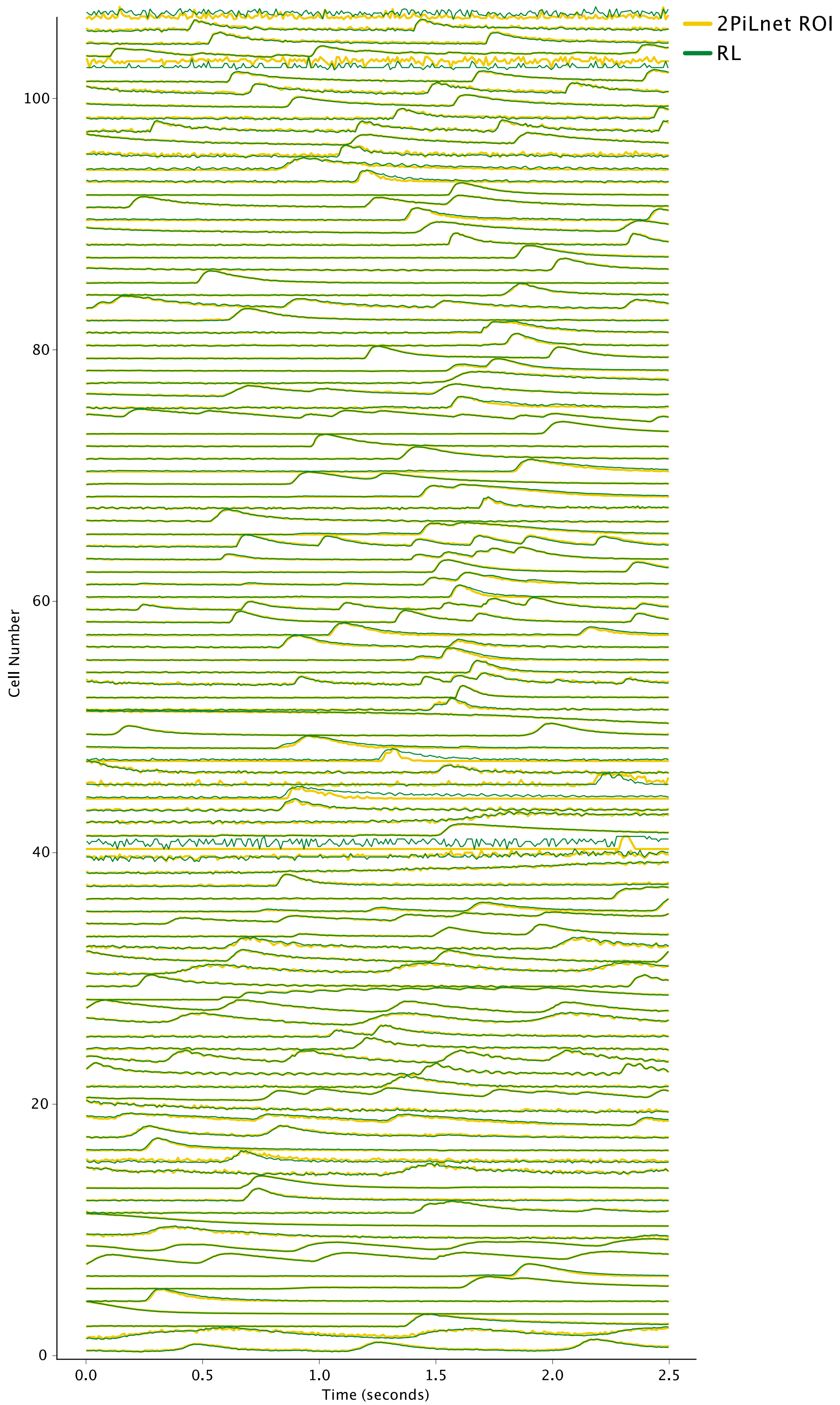

Figure S1: Calcium time series extracted from the same ROIs in volume series reconstructed using 2PiLnet (yellow traces) and 8-iteration RL deconvolution (green traces).

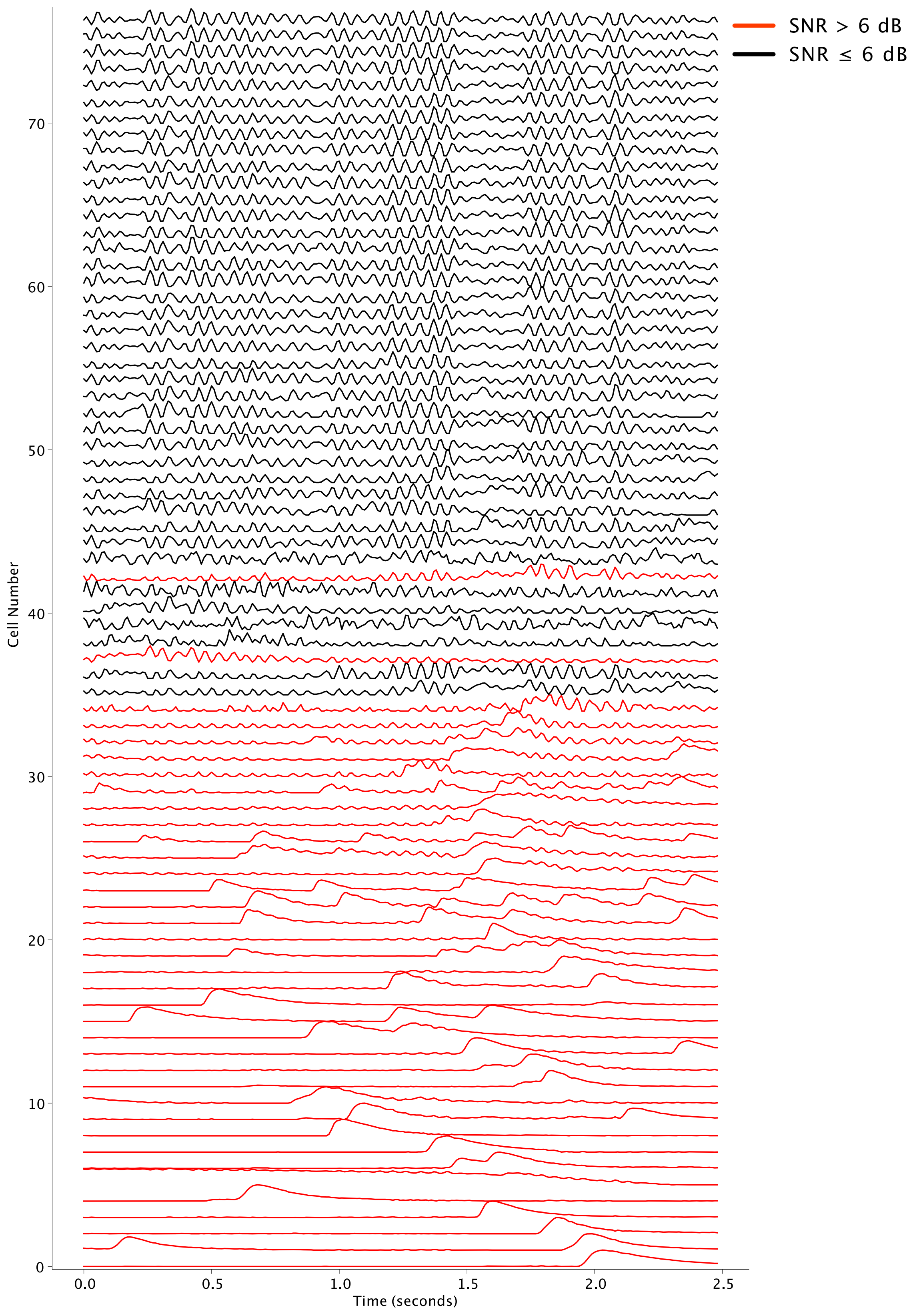

Figure S2: SNR threshold for SID time series inclusion. Due to noise and ringing, only SID-derived time series with SNR > 6 dB (red traces) are considered in SNR and crosstalk analyses.

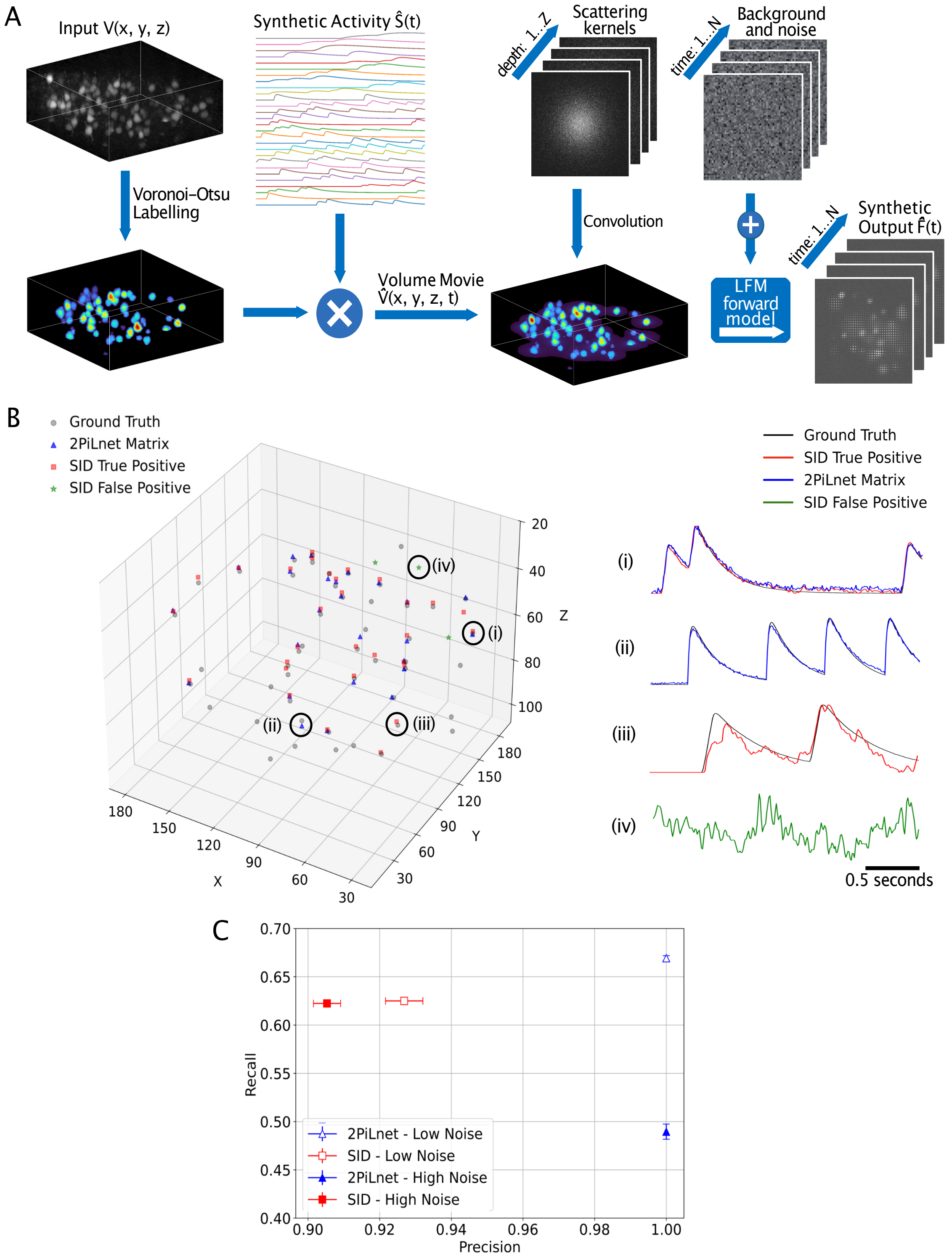

Figure S3: Comparison of 2PiLnet and SID on simulated LF calcium videos. (A) Workflow for generating synthetic LF calcium videos with scattering and noise. (B) Centroids of extracted time series (left) and example time series from neurons detected by (i) both 2PiLnet and SID, (ii) 2PiLnet only, (iii) SID only, and (iv) a false positive detected by SID. (C) Recall and precision as mean and standard error for 8 simulation runs with different Poisson noise seeds. Due to false positive detection, SID shows lower precision than 2PiLnet in both low (Poisson scale 20,000) and high (Poisson scale 255) noise conditions ($\Delta = -0.073 \pm 0.005$ and -0.095 ± 0.004, respectively). Recall is slightly higher for 2PiLnet in low noise ($\Delta = 0.045 \pm 0.003$) and lower in high noise ($\Delta = -0.133 \pm 0.007$) than SID (all Wilcoxon signed-rank $p = 0.0078$).

Supplementary Methods

Evaluation of 2PiLnet and SID on simulated calcium light field videos

The performance of 2PiLnet and Seeded Iterative Demixing (SID) on scattering data is evaluated through synthetic calcium LF videos based on recall and precision. The synthetic LF videos are generated through the process shown in Figure S3A.

We start with an empty volume with dimensions corresponding to typical experimental imaging conditions, which is then populated with neurons. The fluorescence and spatial positions of the synthetic neurons are based on experimental data. Specifically, we obtain a high-resolution two-photon scanned volume of neurons transfected with tdTomato, $\boldsymbol{V}(\boldsymbol{x},\boldsymbol{y},\boldsymbol{z})$, which is then segmented with Voronoi-Otsu labelling to obtain 3D neuron positions. This ensures that the brightness and spatial distribution of neurons in the synthetic volume are representative of actual brain tissue. Additionally, we generate time series for a subset of synthetic neurons, $\widehat{\boldsymbol{S}}(\boldsymbol{t})$, using the method outlined in (1). These time series are multiplexed with a randomly selected subset of neurons in the synthetic volume to obtain synthetic volume video, $\widehat{\boldsymbol{V}}(\boldsymbol{x},\boldsymbol{y},\boldsymbol{z},\boldsymbol{t})$.

We simulate the scattering effect of brain tissue by convolving each z-axis slice in the synthetic volume with a depth-dependent PSF kernel obtained through Monte Carlo simulation. We follow the procedure outlined in (2) to simulate the propagation of photons through a slab of scattering tissue from $0 \leq z \leq z_h$. We use a characteristic scattering length of μ = 10 mm^{-1} and a forward scattering anisotropy of g = 0.86 for the Henyey-Greenstein phase function (3). This convolution step is repeated for each frame in the synthetic volume video to obtain a scattering synthetic volume video. We then make use of the physics-based forward model introduced in (4) to generate LF video, $\widehat{\boldsymbol{F}}(\boldsymbol{t})$, which accounts for the imaging optics of the system. Additionally, uniform background fluorescence (10% of the dynamic range), Gaussian noise (σ = 0.001), and Poisson noise are added on a frame-by-frame basis. For “low noise” the Poisson scale is 20,000. For “high noise” the Poisson scale is 255.

The performance of 2PiLnet is evaluated using recall and precision metrics. Since we make use of synthetic data, we can match the spatial positions of recovered neurons to that of ground truth neurons. This allows us to identify the number of true positives (TP) (a recovered neuron is matched to a ground truth neuron), false positives (FP) (a neuron is recovered where no neuron exists in ground truth), and false negatives (FN) (ground truth neurons that were missed). Precision and recall are calculated as follows (5):

$$\boldsymbol{precision} = \frac{\boldsymbol{TP}}{\boldsymbol{TP}+\boldsymbol{FP}}$$

$$\boldsymbol{recall} = \frac{\boldsymbol{TP}}{\boldsymbol{TP}+\boldsymbol{FN}}$$

1. Y Zhang, et al., Fast and sensitive gcamp calcium indicators for imaging neural populations. *Nature* 615, 884–891 (2023).
2. P Quicke, et al., High speed functional imaging with source localized multifocal two-photon microscopy. *Biomed. Opt. Express* 9, 3678–3693 (2018).
3. AN Yaroslavsky, et al., Optical properties of selected native and coagulated human brain tissues in vitro in the visible and near infrared spectral range. *Phys. Medicine Biol.* 47, 2059 (2002).
4. H Verinaz-Jadan, et al., Physics-based deep learning for imaging neuronal activity via two-photon and light field microscopy. *IEEE Transactions on Comput. Imaging* 9, 565–580 (2023).
5. DD David L. Olson, *Advanced Data Mining Techniques*. (Springer Berlin, Heidelberg), (2008).

Movie S1 (separate file). Movie of 2PiLnet (right), 8-iteration RL deconvolved (middle), and 1-iteration RL deconvolved (left) volumes, related to Figure 2. Playback at 0.5x real speed.
